# Next RNA Therapeutics: The Mine of Non-Coding

**DOI:** 10.3390/ijms23137471

**Published:** 2022-07-05

**Authors:** Sabrina Garbo, Rossella Maione, Marco Tripodi, Cecilia Battistelli

**Affiliations:** 1 Department of Molecular Medicine, Faculty of Pharmacy and Medicine, Sapienza University of Rome, Viale Regina Elena 324, 00161 Rome, Italy; sabrina.garbo@uniroma1.it (S.G.); rossella.maione@uniroma1.it (R.M.); 2 National Institute for Infectious Diseases “L. Spallanzani”, Via Portuense 292, 00149 Rome, Italy

**Keywords:** ncRNAs, RNA therapeutics, delivery strategies

## Abstract

The growing knowledge on several classes of non-coding RNAs (ncRNAs) and their different functional roles has aroused great interest in the scientific community. Beyond the Central Dogma of Biology, it is clearly known that not all RNAs code for protein products, and they exert a broader repertoire of biological functions. As described in this review, ncRNAs participate in gene expression regulation both at transcriptional and post-transcriptional levels and represent critical elements driving and controlling pathophysiological processes in multicellular organisms. For this reason, in recent years, a great boost was given to ncRNA-based strategies with potential therapeutic abilities, and nowadays, the use of RNA molecules is experimentally validated and actually exploited in clinics to counteract several diseases. In this review, we summarize the principal classes of therapeutic ncRNA molecules that are potentially implied in disease onset and progression, which are already used in clinics or under clinical trials, highlighting the advantages and the need for a targeted therapeutic strategy design. Furthermore, we discuss the benefits and the limits of RNA therapeutics and the ongoing development of delivery strategies to limit the off-target effects and to increase the translational application.

## 1. Introduction

In the last decades, growing attention pointed to the development of innovative therapeutic strategies that were based on new scientific findings. Specifically, the rapid development of RNA-based approaches that were used in basic research for scientific purposes inspired the application of these strategies to clinical research. More recently, thanks to the increased interest in ncRNAs (e.g., miRNAs, lncRNAs, circRNAs) as regulators of gene expression in pathophysiology (e.g., cancer, neurodegenerative diseases, cardiopathies [1]), the potential approaches that were based on the translation of these molecules into a therapeutic perspective grew exponentially. While the common use of small molecules represents a fundamental approach to counteract proteins’ activities in pathological contexts (e.g., by selectively inhibiting active sites or competing with the interaction with the substrate [2,3,4,5]), the development and optimization of RNA-based strategies nowadays represents a further promising route. Here, we report an overview of the principal classes of therapeutic ncRNAs that are used in clinical practice, with a focus on the advantages of using this kind of molecule in therapy the need to optimize and increase knowledge in this field for future clinical application.

## 2. RNA Interference (miRNAs, siRNAs and ASOs)

### 2.1. miRNAs

**General concepts: miRNAs** are a class of short non-coding RNAs that are characterized by a length of 20–22 nt, with a fundamental functional role in regulating gene expression at a post-transcriptional level. Their mechanism of action is exerted by their ability to interact with the RNA-induced silencing complex (RISC complex) and, in turn, to target a specific mRNA in order to block its translation and/or induce its degradation. miRNAs, natural short antisense RNAs, are essential for the correct gene expression program during development and organogenesis; indeed, a global decrease in miRNA expression is lethal [6,7,8,9].

miRNAs are transcribed into the nucleus as precursor molecules (pri-miRNAs), which can be more than 1000 nt long and are processed in 60–120 nt-long pre-miRNAs by Drosha/DGCR8. Exported by exportin-5 into the cytoplasm, these molecules are processed via cleavage by Dicer in order to obtain a short double-strand miRNA, which in turn is recruited into the RISC complex, where a selection of one single strand occurs and interacts with Argonaute (Ago) proteins to target the 3′UTR mRNAs [6].

In contrast to siRNA molecules (see below) and other non-coding RNAs, they are natural and naturally able to be processed and to function afterward. Depending on the degree of complementarity, miRNAs can induce mRNA degradation (perfect match) or translational inhibition (imperfect match). In addition, their activity is not limited to a single messenger RNA, but they usually target several mRNAs, often included in the same molecular pathway. This amplifies the miRNA function effects, since they repress the expression of several interconnected targets simultaneously; however, this also implies that miRNA activity is promiscuous.

**The therapeutic potential** of miRNAs is based on the knowledge of specific miRNA families involved in several biological processes [10] that are dysregulated in different pathologies, such as cardiovascular diseases [11,12,13,14,15], viral infections [16,17], metabolic diseases [18], diabetes [19], stress-related diseases [20,21,22] and cancer [23]. For this reason, it is conceivable that a molecular therapy takes advantage of their use. Indeed, thanks to biotechnological advancements, it is possible to benefit from miRNA mimics (miRNA-like dsRNAs), in order to boost some miRNA expression and function; at the same time, antagomiRs can be used as miRNA inhibitors in order to block the function of specific miRNAs [24,25]. The use of these small molecules, previously developed for basic and applied research to identify miRNA-specific functions, now represent a selective approach for translational/therapeutic applications.

**Ongoing approaches:** At present, the approval of miRNA therapeutics in clinical practice needs further steps of effectiveness validation; however, several miRNAs are under clinical trials for the treatment of different diseases. As examples, cobomarsen targets miR-155 for the treatment of blood cancers, remlarsen mimics miR-29 in keloids treatment and MRG110 is an antagomir for miR92 that is proposed for tissue repair [26].

**Conclusions and future perspectives**: miRNA-based therapy is a well-recognized, exciting and significant approach that holds promise for next generation drugs than can reach specific targets and sites. Several efforts are incoming for the development of therapeutic miRNAs with advanced absorption, distribution, metabolism and elimination abilities. Moreover, great attention is needed for the delivery of specifically localized miRNAs to selected target sites. This delivery optimization should efficiently improve the effectiveness of therapeutic miRNAs.

### 2.2. siRNAs

**General concepts:** Short interfering RNAs (**siRNAs**) are double-stranded short RNAs that are involved in the RNA interference pathway. These molecules are a product of evolution as a cellular defense from viral RNAs. As described for miRNAs, one strand of the siRNA is complexed with AGO2 in the formation of the RISC complex, and this interaction allows the cleavage of a specific transcript.

In contrast to miRNA processing, prior to Dicer activity, siRNAs are double-stranded RNAs of 30–100 nucleotides, entirely complementary to the target mRNA.

siRNAs, discovered in plants, invertebrates, and zebrafish, are not naturally produced in mammals, except in murine germline cells [27].

**Therapeutic potential:** Concerning the biotechnological application, synthetic siRNAs can be designed to obtain a potent knockdown of a specific gene. This approach, routinely applied to the characterization of gene functions in basic biology, represents, at present, a translational strategy to selectively impair genes that are causal to several diseases.

**Ongoing approaches:** As reported in Table 1, several siRNAs are currently used in therapy or are under clinical trials to treat different pathologies.

**Conclusions and future perspectives:** one of the major issues regarding therapeutic siRNAs concerns their stability, safety and specificity. The accumulation of these molecules should reach a high level of biocompatibility in order to not compromise organ functionality. Moreover, the combination of several siRNAs may help in the targeting of complex pathways with the intent to sensitize the target site and allow the treatment of patients with similar but not identical clinical pictures. This is in line with a perspective of a personalized medicine.

### 2.3. ASOs

**General concepts:** Antisense oligonucleotides (ASOs) are short, single-stranded RNA or DNA oligonucleotides that also provide gene silencing. They can be naturally produced by the cells or artificially synthesized, and they have the ability to target a specific transcript through the Watson and Crick base complementarity. They can work by inducing transcript degradation and altering mRNA processing, blocking RNA translation, preventing ribosome attachment [28].

**Therapeutic potential:** There are several ASOs in clinical trials, especially targeting mRNAs that are causal of several diseases [29]; however, the setup of ASOs for ncRNAs (see below) opens the way for a selective and efficient ncRNA druggability.

**Ongoing approaches:** Among ASOs that are applied in clinical management (See Table 2), Eteplirsen is a morpholino-oligomer antisense that is used to bind to dystrophin pre-mRNA in order to obtain exon 51 exclusion by an exon skipping event, with the purpose to restore the correct reading frame of the dystrophin transcript [30]. SMA (spinal muscular atrophy) is an autosomal recessive disease that is characterized by different mutations such as a C->T within the exon 7, causing an exclusion that drives the production of a truncated form of the survival of the motor neuron 2 (SMN2) protein. The use of an ASO (Nusinersen) is designed to bind to the SMN2 pre-mRNA and promote the inclusion of exon 7 for the restoration of the full-length protein [31].

**Conclusions and future perspectives:** The breakthroughs in the field of RNA ASOs make them good candidates use in the treatment of distinct pathologies. The main concern regarding ASOs is the delivery of these therapeutics; specifically, their protection should be optimized to maintain their integrity (i.e., in the gastric and intestinal tract) and to enhance their absorption.

## 3. lncRNAs

**General concepts:** lncRNAs (long non-coding RNAs) are non-coding RNAs with a length of more than 200 nt [32]. They are characterized by the absence of an open reading frame (ORF) and can be subjected to cap addition at 5′, polyadenylation at 3′ and splicing events, as for mRNAs [33]. In recent decades, the identification of several lncRNAs involved in physio-pathological conditions [34,35,36,37] aroused a great deal of interest in this class of molecules, not only as possible targets, but also as therapeutic RNAs. The activities of the few lncRNAs that have been functionally characterized may occur in both the nucleus and the cytoplasm, where they interact with DNA, different RNAs, and proteins. lncRNAs can sponge several miRNAs and proteins in order to delocalize and degrade them [38], mediate the interaction between messenger RNAs and polysomes in the cytoplasm [39], act as molecular scaffolds to allow the assembly of ribonucleoprotein complexes [40] and behave as epigenetic molecular enhancers or repressors in the nucleus through the enrollment of epigenetic modifiers [41].

**The therapeutic potential:** Based on their diversified roles, long non-coding RNAs have been considered as suitable targets to be used for therapy. An example of ncRNA-based therapeutic molecules that can recognize and antagonize lncRNA expression is represented by the use of siRNAs and antisense oligonucleotides (ASOs) [42].

In addition, the potential of lncRNAs is exerted by their use as therapeutic molecules.

For example, an approach to target specific mRNAs through lncRNAs is based on the use of SINEUPs, a new class of antisense lncRNAs that can increase the translation of specific mRNAs. These molecules are characterized by two main domains: one is the SINE element, which is the effector domain for the increase in translation activity; the other is the AS (AntiSense) region, which is able to target the specific mRNA through base pairing [43].

With respect to RNA-based therapies to counteract lncRNA function, an approach was recently designed to counteract EMT (epithelial to mesenchymal transition) that was based on the use of a dominant-negative form of the lncRNA HOTAIR [44]. This first reported lncRNA-based approach to counteract EMT exploits a module of the lncRNA HOTAIR (HOTAIR-sbid) that includes the SNAIL-binding domain but is depleted of the EZH2-binding domain. Mechanistically, HOTAIR-sbid acts as a dominant-negative of the endogenous HOTAIR function in regulating SNAIL activity, since it binds to SNAIL but is unable to mediate the interaction between the sequence-specific transcriptional factor SNAIL and the general chromatin modifier EZH2. This, in turn, impairs the H3K27me3/EZH2-mediated repression of epithelial SNAIL-target genes. Functionally, HOTAIR-sbid expression impairs cellular motility, invasiveness, anchorage-independent growth in HCC cells, and responsiveness to TGFβ-induced EMT [44].

**Ongoing approaches:** Concerning lncRNAs, at present, the principal application regards the modulation of their expression in order to obtain oncogenic lncRNAs knockdown (by RNA interference approach), or oncosuppressor lncRNA overexpression; therefore, the use of these molecules per se is a hot topic that requires development. With respect to SINEUPs, this class of both natural and synthetic lncRNAs shows a significant applicability in therapeutic approaches and has been described as a powerful strategy to treat haplo-insufficiencies.

**Conclusions and future perspectives:** lncRNAs are certainly considered as pivotal elements in the development of different pathologies, including cancer. Based on these findings, the development of more effective therapeutics involving this class of molecules represents an emerging challenge. The development of more specific gene editing approaches, as well as the design and characterization of lncRNA-competing molecules, limiting the off-target effects, should be considered in the perspective of lncRNA-based therapeutic approaches.

## 4. circRNAs

**General concepts:** circRNAs (circular RNAs) are non-coding RNAs that are obtained through a biogenesis mechanism, characterized by back splicing events. Back-splicing requires a flanking intron with a downstream 3′ donor site and upstream 5′ acceptor sites [45]. They are closed single-stranded transcripts and, thanks to their circular structure, they have greater stability with respect to linear RNAs; for this reason, they can have a longer half-life and could be useful for therapeutic intents. Their circular structure protects them from deadenylating events and other processes of RNA degradation [46,47,48,49].

**The therapeutic potential** of circRNAs to act as molecular sponges for miRNAs could be very interesting in order to delocalize oncogenic miRNAs or miRNAs that are causal to several diseases’ pathways. However, other interesting functions have been described for circRNAs, including their ability to bind and sponge proteins (RNA-binding proteins [50], transcriptional factors [51,52,53,54], and chromatin remodeling enzymes [55,56,57]), increase proteins translation, modify proteins at a post-translational level, and transcriptionally regulate gene expression [58,59].

Finally, based on the described ability of circRNAs to be translated [60,61], this class of molecules could be used to produce RNA vaccines. In this context, we can mention that circRNAs can be used to produce polypeptide-like antigens, receptors, or other functional proteins.

**Ongoing approaches:** A recent application of this strategy concerns a coding circRNA that is used as a vaccine against Sars-CoV2 [62]. This approach can induce a potent humoral and cellular immunity and elicits a proportion of neutralizing antibodies that have a more durable antigen production with respect to the 1mΨ-modified mRNA vaccine. The necessity to use circRNA vaccines is linked to the need of an RNA vaccine that is more thermally stable, with an enhanced efficacy and translation, as well as more stable in both naked and encapsulated form. Thus, the development of circRNA-based molecules could be useful for this intent.

**Conclusions and future perspectives:** Due to the wide spectra of activities that are attributable to circRNAs and their stability (due to their structure), circRNA targeting or impairment represent a potentially powerful strategy to impair several diseases’ onset and progression. CircRNAs are generally expressed in a tissue-specific pattern; however, limiting the off-target effects represent an open field of investigation. To this issue, the specific vehiculation of synthetic circRNAs or siRNAs targeting the junction sequence may provide substantial benefits for patients. Moreover, their modulation in vivo, based on the needed knowledge in this field, will advance the applicability of circRNA therapeutics.

## 5. Ribozymes

**Figure 1:**
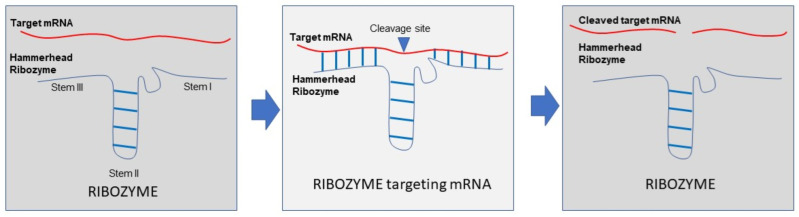
Schematic representation of hammerhead ribozymes mechanism of action.

**General concepts:** Ribozymes are RNA molecules that are characterized by catalytic activity such as proteins. They are able to cleave target RNAs and thus impact gene expression [63] and allow gene manipulation. This class of molecules is characterized by two domains: one is the sequence that recognizes a region of the target RNA by base complementarity, and the other is the catalytic domain [64]. The best-characterized and studied are the hammerhead ribozymes. The minimal sequence of these ribozymes consists of a well-conserved core region of fifteen nucleotides that are flanked by three stems with a helical structure, and their activity requires an interaction between specific sequences on the first and second stem [65]. Their folding allows their catalytic function (splicing or cleavage of other RNA molecules) in a sequence-specific manner through base-annealing of the ribozyme sequence with the regions flanking the cleavage site of the target RNA (Figure 1).

**The therapeutic potential** of ribozymes resides in their ability to selectively recognize a specific RNA (e.g., an RNA associated with a disease) and to cleave it (with the consequent abrogation of RNA translation); these specific features make this class of molecules a promising, innovative and specific approach to be used in therapy as an RNA drug. Moreover, once the target RNA is cleaved, the ribozyme can detach from the substrate and can go on to bind and cleave other targets. Furthermore, ribozymes can be artificially engineered in order to have cleavage activity in *cis* or in *trans*, and to be allosterically activated by specific effector molecules [66].

**Ongoing approaches** include Angiozyme, a synthetic 35-nucleotide-long ribozyme that is used in clinics to target the VEGFR mRNA to impair angiogenesis and tumor progression in renal carcinoma [67]. Moreover, ribozymes that are used for infective disease treatment have been reported, such as Heptazyme, which is able to specifically cleave the HCV RNA genome [63,68,69]. With respect to other ribozymes that are used as a gene therapy strategy to prevent HIV replication in Cd4+ T-cells, some studies indicated that they are effective as single agents, as well as in combination with other therapies [70,71].

**Conclusions and future perspectives:** Ribozymes-based therapeutic approaches seem to be promising agents as alternative or adjuvant to chemotherapy. Moreover, their ability to catalytically inactivate many specific target RNAs and to maintain a higher-grade of stability with respect to ASOs, together with their negligible toxicity, confer them the potential to be applied to the patients’ management. At present, a challenge in the use of ribozymes as therapeutic molecules is centered on the need to improve their stability, activity and delivery to specific cells.

## 6. CRISPR-CAS System on RNA

**General concepts:** In recent years, the CRISPR-Cas9 system has been significantly improved as a strategy of genome editing, alongside the emergence of some concerns regarding clinics, as well as scientific controversies.

Based on this technology, the first core member of the system, a non-coding RNA that is represented by the guide RNA (gRNA), specifically targets the portion of genomic DNA to be engineered, while the Cas9, the enzyme component, cuts the DNA at specific sites that are complementary to the gRNA sequence.

A CRISPR-Cas system on RNA is also being developed. Differentially to Cas9, Cas13 only has target activity on RNA molecules, conferring to this strategy the possibility to manage and edit RNA for therapeutic purposes [72,73]. RNA editing shows several advantages with respect to genome editing; while Cas9 can display some long-term and irreversible off-target effects, an RNA targeted system is safer, and CRISPR-Cas13 could be useful for RNA knockdown and the regulation of gene expression at a post-transcriptional level. A further approach is represented by the CRISPR-dCas9. In this case, the strategy is based on using a catalytic inactive Cas9 (dead Cas9) that can target RNA molecules to modulate ncRNA function, target splicing processes to induce RNA decay, or target RNA translation inhibition. Moreover, as a further update of the CRISPR-Cas system, it is possible to obtain fusion proteins between Cas13 and an enzyme or a protein of interest with the intent to control splicing, to regulate translation and stability, or to edit some chemical modifications on specific RNAs.

**The therapeutic potential** of the use of a guide RNA represents, per se, a strategy to use an RNA molecule.

**Figure 2:**
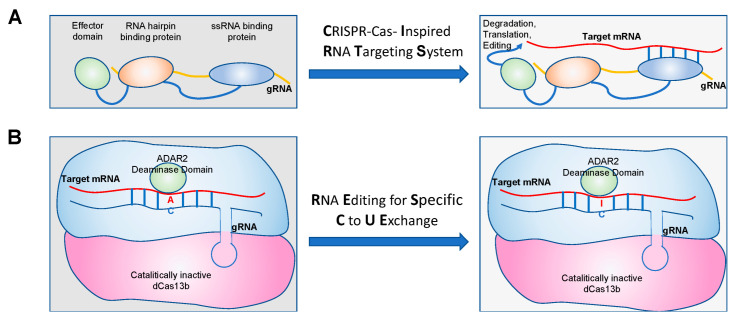

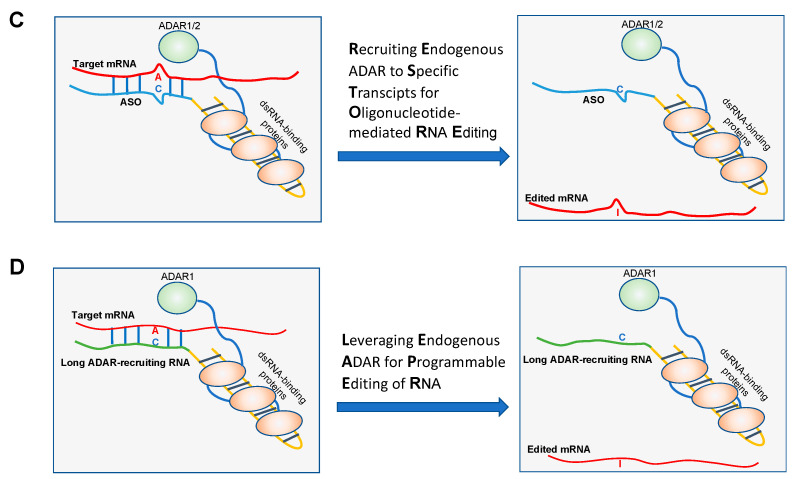
Schematic representation of (**A**) CIRTS, (**B**) RESCUE, (**C**) RESTORE, and (**D**) LEAPER RNA editing approaches.

This approach has been applied at present to Adenosine deaminase acting on RNA (ADAR)-mediated RNA editing and has inspired further programmable RNA-guided machines for potential translational applications. In this regard, ADAR-mediating RNA editing, such as CIRTS (CRISPR-Cas-inspired RNA targeting system); RESCUE (RNA editing for specific C to U exchange); RESTORE (recruiting endogenous ADAR to specific transcripts for oligonucleotide-mediated RNA editing); and LEAPER (leveraging endogenous ADAR for programmable editing of RNA) have been developed and validated. The CIRTS system (Figure 2A) is composed of a gRNA, an ssRNA-binding protein, and a hairpin RNA-binding protein, which is linked to an effector protein such as the human adenosine deaminase for the transcripts containing G to A mutations in order to correct them. RESCUE (Figure 2B) is composed of a dead Cas13 (non-catalytic Cas13) that is fused to the human deaminase domain of ADAR2 in order to induce RNA editing. RESTORE (Figure 2C) is formed by a modified ASO with a domain that gives specificity to target RNA and a domain of recruitment of endogenous ADAR enzymes. LEAPER (Figure 2D) is a strategy that is composed of an ADAR-recruiting RNA that anneals with the target transcripts with an A-C mismatch, and the double-stranded substrate is recognized by the ADAR1 enzyme [74]. All of these approaches could be applied to correct RNA level point mutations (G-A) that occur at genomic sites and that are causative of disease onset.

Cas13 editing can also be applied to add chemical modifications to specific RNAs for therapeutic purposes [75], in order to promote the stability of targeted mRNAs through the recruitment of enzymes that are responsible for RNA post-transcriptional modifications. In this regard, the METTL3-METTL14 writing complex that is fused to the Cas13 is able to specifically modify (i.e., m6A modification) selected target RNAs [75].

**Ongoing approaches:** The deadCas13 can edit RNAs at different levels, representing a powerful strategy to be applied to several diseases, including neurodegenerative diseases that are caused by the triplet repeat expansion. One of the most interesting aspects of this approach is that Cas13 and dCas13 can target numerous RNAs or different regions of the same RNA at the same time, representing an efficient tool for RNA-based approaches.

**Conclusions and future perspectives:** The Cas13-based approach represents a knockdown strategy that is more specific and efficient than the RNA interference and with no detectable off-target effects. Moreover, by localizing the enzyme into the nucleus (in a fused form with a nuclear localization sequence), it can significantly interfere with nuclear RNAs. Starting from the promising data that can be obtained from basic research, the development of these effective, specific and multitarget approaches to be applied to oncogenic RNAs could be desirable for complex diseases management, at first for in vitro study, and then for in vivo applications.

## 7. Aptamers

**General concepts:** RNA aptamers are artificial structured sequences that can specifically bind to different classes of molecules and can be used in therapy for the treatment of pathological conditions, but also as diagnostic tools. They can be used to impact gene expression, and it is also possible to specifically design aptamers against specific ligands to be used as RNA antibodies [76]. The SELEX (systematic evolution of ligands by exponential enrichment) approach makes it possible to optimize them by a rigorous selection in vitro [77]. The SELEX technology comprises three fundamental steps: selection, partitioning, and amplification [78]. Initially, a library of RNA oligonucleotides is synthesized with some unique sequences that are flanked by two primer binding sites necessary for the following PCR amplification. The oligonucleotides are then incubated with target molecules; the unbound ones are eliminated, and the bound ones are retrotranscribed into cDNA and subjected to PCR amplification. Several rounds of positive and negative selection are needed to increase specificity. The better ones are then subjected to sequencing, and other binding affinities are evaluated [79].

**The therapeutic potential** of aptamer technology to impact gene expression is represented by the insertion of an RNA aptamer into the 5′UTR of mRNAs; upon ligand binding, there is a conformational switch that does not allow ribosome scanning at 5′UTR, with a consequent substantial decrease in translation activity [80].

Moreover, ligand-bound aptamers could be used to impact alternative splicing or exon skipping events through RNA conformational switches that impact the regulation of splicing machinery activity [81]. RNA aptamers are also useful to prevent RNA interference; for this purpose, an aptamer-based strategy is represented by adding an RNA aptamer downstream of a miR-target site at 3′UTR of an mRNA. In this case, after the binding with the ligand, the conformational change prevents the RNA interference by the miRNA [82].

**Ongoing approaches:** As for the other mentioned RNA-based approaches, some aptamers have also shown encouraging readouts in terms of cellular response to these therapeutic molecules (Table 3). Indeed, Gint4.T, an RNA aptamer that can bind and downregulate PDGFRβ, promotes a decrease in proliferation and migration in glioblastoma multiforme cells [83], while CL4, an RNA aptamer against EGFR, demonstrated an encouraging effect against EGFR-positive tumoral cells [84].

**Figure 3:**
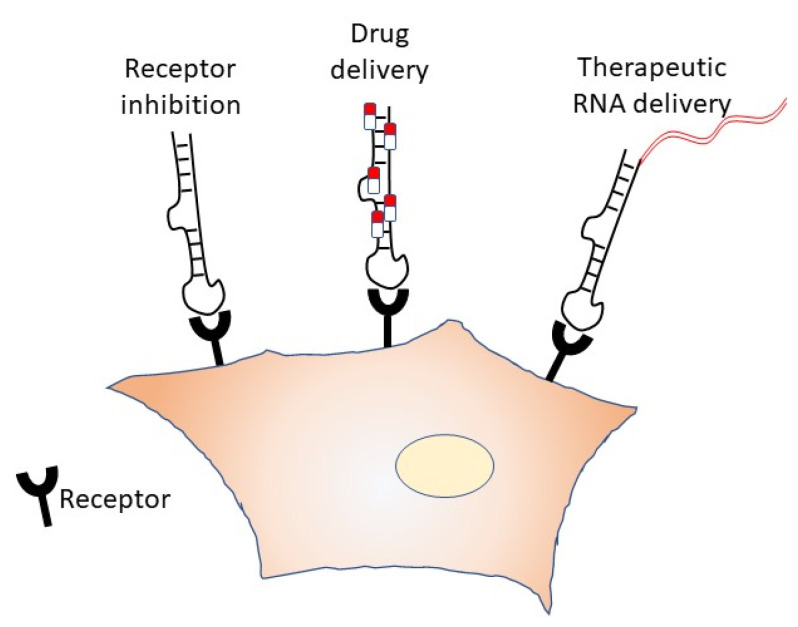
Schematic representation of aptamers engineering on receptor inhibition, drug delivery, and therapeutic RNA vehiculation.

With respect to the possible engineering of RNA aptamers, they could also be used as vehicles for drugs through conjugation with other molecules, or to other classes of RNAs [85] (Figure 3); this is obtainable thanks to the receptor-mediated internalization of modified-aptamers [86]. This approach is fascinating, since it could vehiculate specific molecules to selected targets.

Finally, aptamers can also be used for diagnostic purposes; indeed, their binding ability can be exploited to fluorescently stain patient tissues for a specific disease marker (as RNA antibodies) [87].

**Conclusions and future perspectives:** The potentiality of aptamers in therapy is based on their specific features of low immunogenicity, safety, stability at room temperature, possibility to be conjugated to other small molecules, and customization. At present, the completion of the molecular mechanism-elucidating aptamers’ mechanism of action is ongoing, but the principal issue to be addressed remains their delivery. Thus, the development of carriers that can drive aptamers to specific sites remains an open field of investigation. The drug delivery of modified aptamers could allow to reach specific targets, and their synthetic origin warrants at first the reproducibility of the experimental data, and then of the clinical application in advanced stages of experimentation and clinical practice.

## 8. State of the Art in the Clinics

Several companies are currently investing in the research and development of new RNA drugs to cure different diseases (Alnylam Pharmaceuticals, Moderna, Ionis Pharmaceuticals, Gotham, BioNTech Therapeutics, CureVac). The importance of the development of the two mRNA-based vaccines against COVID-19, mRNA-1273 (Moderna) [88], and BNT162b2 (Pfizer-BioNTech) [89], during these years represents the potentiality of the RNA-based therapy.

Beyond the use of messenger RNAs for therapeutic purposes, several non-coding RNA molecules have been approved by the FDA (Food and Drug Administration) or are currently in clinical trials.

Great opportunities have been given to siRNAs and antisense oligonucleotides (ASOs). Approved siRNAs such as patisiran [90] in 2018 for the treatment of transthyretin amyloidosis, and givosiran [91] in 2019 to treat acute hepatic porphyria, should be mentioned. Nusinersen [92] (Spinraza) is an ASO that was approved by the FDA in 2016 to induce exon skipping for the treatment of SMA (spinal muscular atrophy); Eteplirsen [93] (Exondys 51), and Golodirsen [94] (Vyondys 53) are antisense oligonucleotides that were approved for the treatment of Duchenne muscular dystrophy in 2016 and 2019, respectively.

Moreover, it is worth mentioning the first clinical trial for the delivery in vivo of components of the CRISPR-Cas9 through viral vectors, with the challenge to use RNA-LNP (RNA-lipid nanoparticles) as delivery systems in animals. One example of this approach is represented by the correction of a splicing mutation of fumarylacetoacetate hydrolase [95].

With respect to cancer, there are some RNA drugs in clinical trials targeting central genes that are responsible, among others, for cell-cycle regulation, cell proliferation, and apoptosis, as reported in Table 4.

However, it seems that there was more success in the development and translation into clinics of RNA drugs for the cure of other kinds of diseases with respect to cancer. This is probably justifiable by their nature; most of these disorders are monogenic, so it is simpler to obtain a successful response, while cancer is a multigenic disease, and several signaling pathways are dysregulated. Based on this evidence, it seems more challenging to find a drug that can counteract cancer, considering the different stages of tumorigenesis, the pleiotropic effects of the treatment, and the heterogeneity that is observed among cells and individuals.

## 9. Delivery Strategies

Among all these classes of RNAs and their applicability to translational approaches, one of the most stimulating topics in this field of investigation is represented by the development of strategies to deliver these RNAs for therapeutic purposes and to enhance their stability and efficiency for the on-target effects, limiting the off-target ones.

One urgent need is represented by the development of strategies to stabilize or to enhance the half-life of these molecules. Due to the high sensitivity of RNAs to RNAses into the serum, chemical modifications seem to be very challenging. RNA is a hydrophilic and negatively charged molecule, so it has difficulties to enter in naked form into the cells because of electrostatic repulsion. This is why it needs some appropriate vehicles to penetrate target cells and gain function [96].

Lipid nanovesicles are small vesicles that recently gained great success as vehicles for RNA vaccines (e.g., COVID-19 vaccines) [97]. They are characterized by the presence of lipids that mimic the cell membrane, and PEG-lipids (polyethylene glycol-anchored lipids) to prevent the interaction with environmental elements [96]. Indeed, the advantage of PEG-lipid addition is the improvement of circulation time for liposome-encapsulated (LNP) drugs and the reduction in non-specific uptakes. Moreover, positively charged lipids enhance RNA encapsulation thanks to electrostatic interactions.

With respect to extracellular vesicles, these are natural membrane-enclosed particles that are released from the cells with a high impact on physiology and pathology through their important role in intercellular communication. Indeed, extracellular vesicles contain proteins, enzymes, DNA fragments, and RNA molecules, and they carry out a fundamental role in regulating gene expression in target cells [98]. Extracellular vesicles have the great advantage of low immunogenicity, a low degradation rate, and low cytotoxicity [99], and they are able to pass through biological barriers such as the BBB (blood–brain barrier); therefore, they represent a great opportunity to act as RNA carriers into the brain [100,101,102,103].

Extracellular vesicles can be engineered to enclose specific RNA molecules, such as via electroporation. In this case, this method takes advantage of the porous structure of the vesicles, so the RNA drug is incubated with vesicles, and the application of an electric field allows the passage through [99,100,101,102,103,104].

In addition, it is possible to engineer vesicle-producing cells in order to load them with a specific RNA content and specific surface ligands and receptors [105,106]. In this regard, it has previously been reported that the hnRNP SYNCRIP is a component of the hepatocyte’s miRNAs sorting machinery into the exosomes, thanks to its ability to specifically recognize a common extra-seed motif (hEXO) on miRNAs, enhancing their loading into the EVs [107]. These findings open the way to engineer therapeutic miRNAs by adding the hEXO motif in an extra-seed sequence (chimeric miRNAs) to obtain a specific EV-miRNA cargo that could have a substantial impact on intercellular communication for therapeutic purposes. The advantages of RNA therapeutics are represented by the fact that it is simpler and cheaper to synthesize an RNA molecule instead of proteins or chemical drugs, or an antibody, and it is also possible to more easily target undruggable genes with a small molecule or a protein. The increasing knowledge and application of both natural and artificial RNA chemical modifications boosts the possibility of stabilizing and improving the hemi-life of RNA drugs with the intent of obtaining long-term effects. In addition, the use of these molecules in encapsulated form within vesicles means they are more stable and shielded by RNAses and makes it possible to improve the on-target delivery on specific cells and tissues.

## 10. Conclusions

The successful output that was obtained during these years encouraged the scientific community more deeply explore the field of RNA. Looking to the past, we are beyond the doors of the RNA therapeutics era, and several goals have been met [26,108].

Specifically, at present, several RNA therapeutics have been designed and some of them are under clinical trials or being utilized in therapy (e.g., siRNAs, ASOs, aptamers) for different diseases (e.g., cardiovascular pathologies, metabolic diseases, stress-induced pathologies, infectious diseases, cancer).

Nevertheless, there is an urgent need to take advantage of other biological properties of non-coding RNAs, in order to develop strategic alternatives to perturb pathological processes. The great advantages of RNA-based therapies seem to reside in their specificity, low toxicity and ability to act in combination to target complex pathways. Furthermore, some ncRNAs are characterized by a high safety and stability in naked or encapsulated form, representing added value to their applicability. These aspects hold promise for more effective therapeutics and personalized medicine.

The new findings in the field of ncRNAs should further clarify their mechanism of action, may identify other classes of ncRNAs, and may propose new approaches for therapeutic applications of RNAs with a high grade of biocompatibility, absorption, distribution and metabolism.

Furthermore, the future development of the RNA-based therapies must aim at limiting the off-target effects of these molecules and optimizing their low immunogenicity, safety, stability and customization.

In conclusion, the most important points of investigation are dictated by the necessity to conceive novel methods to increase the efficiency, specificity and applicability in clinics of these RNA molecules’ delivery. With these intents, the interdisciplinarity among molecular biology, cell biology, chemistry, nanotechnology and immunology seems to be fundamental.

## Figures and Tables

**Table 1:** List of siRNAs used in therapies or in clinical trials with indicated target gene, disease treated, and reference PMID.

Drug	Target	Disease	Reference
ONPATTRO^®^ (patisiran)	Transthyretin	Hereditary transthyretin amyloidosis	PMID: 29972753
GIVLAARI™	Delta aminolevulinic acid synthase 1 (ALAS1)	Acute intermittent porphyria	PMID: 30726693
Lumasiran (ALN-GO1)	Glycolate oxidase	Primary hyperoxaluria type 1	PMID: 27432743
Inclisiran	Proprotein convertase subtilsin/kexin type 9 (PCSK9)	Primary hypercholesterolaemia or Mixed dyslipidaemia	PMID: 33983616
Fitusiran	Antithrombin	Hemophilia A or B	PMID: 33587824
Remlarsen	Extracellular matrix components	Fibroplasia	PMID: 30472058
SLN124	Transmembrane serine protease 6 (TMPRSS6)	β-thalassaemia	PMID: 33942901
SIG12D LODER	KRAS	Pancreatic ductal adenocarcinoma (PDA)	PMID: 24297898
epha2 DOPC (EPHARNA)	EphA2	Several human cancers	PMID: 28265009
QPI-1002	P53	Acute kidney injury	PMID: 29270490
QPI-1007	Caspase 2	Non-arteritic anterior ischemic optic neuropathy, other optic neuropathies (e.g., glaucoma)	PMID: 25054518
Olpasiran	Apolipoprotein A	Cardiovascular disease	PMID: 35027752
Bamosiran	ADRB2	Ocular hypertension and glaucoma	PMID: 24025749
Cemdisiran	Complement 5 mRNA	Paroxysmal nocturnal hemoglobinuria (PNH)	PMID: 33047216
Tivanisiran	TRPV1	Dry eye disease	PMID: 29569947
Pelacarsen	Apolipoprotein A	Hyperlipoproteinaemia	PMID: 34490591
Nedosiran	LDHA	Primary hyperoxaluria (PH)	PMID: 34481803

**Table 2:** List of RNA ASOs used in clinic or under clinical trials for different diseases treatment.

Drug	Target	Disease	Reference
**Eteplirsen**	Dystrophin pre mRNA	Muscular dystrophy	PMID: 21784508
**Nusinersen**	SMN2	SMA	PMID: 29091570
**Fomivirsen**	CMV IE-2 mRNA	Cytomegalovirus (CMV) retinitis	PMID: 12768225
**Mipomersen**	apolipoprotein B mRNA	Familial hypercholesterolemia	PMID: 30526168
**Inotersen**	Transthyretin mRNA	Hereditary transthyretin amyloidosis	PMID: 29972757
**Viltolarsen**	exon 53 of the dystrophin mRNA precursor	Duchenne muscular dystrophy	PMID: 32519222
**Volanesorsen**	APOC3 mRNA	Familial chylomicronemia syndrome	PMID: 31390500
**Sepofarsen**	c.2991+1655A>G variant in the CEP290 mRNA precursor	Leber congenital amaurosis type 10	PMID: 35379979
**AKCEA-TTR-LRx**	Transthyretin mRNA	Hereditary transthyretin-mediated amyloid polyneuropathy	PMID: 33638113
**Alicaforsen**	ICAM-1 mRNA	Inflammatory disorders	PMID: 11890355

**Table 3:** List of aptamers used in clinics or under clinical trials for several human diseases.

Drug	Target	Disease	Reference
**Gint4.T**	PDGFRb	Glioblastoma multiforme	PMID: 24566984
**CL4**	EGFR	Tumor	PMID: 21915281
**BAFF-R-specific-aptamer**	BAFF receptor	B-cell malignancies	PMID: 23470998
**NOX-A12**	CXCL12	Chronic lymphocytic leukemia	PMID: 31097627
**NOX-H94**	Hepcidin	Anemia of chronic inflammation	PMID: 23349391
**Anti-FXII**	Coagulation factor XII (FXII)	Thrombosis	PMID: 34363738
**Macugen (Pegaptanib)**	VEGF	Macular degeneration	PMID: 16935210
**Emapticap**	CCL2	Albuminuria, diabetes mellitus	PMID: 28186566
**REG1**	Factor IX	Acute coronary syndrome	PMID: 22420328
**BT200**	Von Willebrand factor (VWF)	Thrombosis	PMID: 32636459

**Table 4:** List of RNA drugs used in cancer therapies with the indicated target gene, disease treated, and reference PMID.

Drug	Target	Disease	Reference
AEG35156	XIAP	Breast cancer	PMID: 15378029
G3139/oblimersen	BCL2	AML, CML, NHL, prostate cancer, and breast cancer	PMID: 14716145
LErafAON	RAF	Prostate cancer	PMID: 12429653
AZD9150	STAT3	Lymphoma and lung cancer	PMID: 26582900
AZD8701	FOXP3	Advanced solid tumors	PMID: 33208059
AZD4785	KRAS	Advanced solid tumors	PMID: 28615361
siG12D-LODER	G12D-mutated KRAS mRNA	Pancreatic cancer	PMID: 24297898
Prexigebersen (BP1001-A)	GRB2 mRNA	Acute myeloid leukemia, chronic myeloid leukemia	PMID: 34145432
Apatorsen (OGX-427)	HSP27	Prostate cancer	PMID: 24411988
